# Development of a hydroxyflavone-labelled 4554W peptide probe for monitoring αS aggregation

**DOI:** 10.1038/s41598-023-37655-3

**Published:** 2023-07-06

**Authors:** Kathryn J. C. Watt, Richard M. Meade, Tony D. James, Jody M. Mason

**Affiliations:** 1 Department of Life Sciences, University of Bath, Claverton Down, Bath, BA2 7AY UK; 2 Department of Chemistry, University of Bath, Claverton Down, Bath, BA2 7AY UK

**Keywords:** Biochemistry, Biological techniques, Analytical biochemistry

## Abstract

Parkinson’s is the second most common neurodegenerative disease, with the number of individuals susceptible due to increase as a result of increasing life expectancy and a growing worldwide population. However, despite the number of individuals affected, all current treatments for PD are symptomatic—they alleviate symptoms, but do not slow disease progression. A major reason for the lack of disease-modifying treatments is that there are currently no methods to diagnose individuals during the earliest stages of the disease, nor are there any methods to monitor disease progression at a biochemical level. Herein, we have designed and evaluated a peptide-based probe to monitor αS aggregation, with a particular focus on the earliest stages of the aggregation process and the formation of oligomers. We have identified the peptide-probe K1 as being suitable for further development to be applied to number of applications including: inhibition of αS aggregation; as a probe to monitor αS aggregation, particularly at the earliest stages before Thioflavin-T is active; and a method to detect early-oligomers. With further development and in vivo validation, we anticipate this probe could be used for the early diagnosis of PD, a method to evaluate the effectiveness of potential therapeutics, and as a tool to help in the understanding of the onset and development of PD.

## Introduction

Despite being the most common motor neurodegenerative disease, there is currently no way to conclusively diagnose Parkinson’s disease (PD), and this is especially true in the earliest stages of the disease. At present, the main method of PD diagnosis is through the observation of motor symptoms—such as motor rigidity, resting tremor and postural instability—however, these symptoms are only present in the later stages of the disease^1^. The inability to conclusively diagnose PD in the earliest stages of aggregation—a key diagnostic for the disease—is one of the reasons why no disease-modifying treatment is currently available. By not being able to identify individuals with early (prodromal) forms of the disease, they are not able to be recruited for clinical trials at a stage where the treatments are designed to be most effective^2–4^. Furthermore, once a clinical trial is underway, there is no way to clinically measure the effectiveness of treatments at a biochemical level^3,4^. Consequently, many potential treatments fail due to ineffective methods to diagnose and track disease progression.

Therefore, the design and development of methods to accurately monitor biomarkers that correlate to disease progression are highly desirable. To be able to predict, diagnose, and monitor disease progression at a biochemical level would greatly assist in improving the diagnosis of PD, and hence would also enable improved treatments. Several potential biomarkers have been identified in PD, however, αS is the most highly validated to date due to its association with familial PD and its presence in Lewy bodies^5–7^. In addition to being extremely relevant at a biochemical level (for example, through the association of αS oligomerisation and cell toxicity), αS is a highly desirable biomarker due to it being readily excreted into extracellular space and can be identified in a wide range of biological fluids including cerebrospinal fluid (CSF), blood, saliva, and tissue samples such as the gastrointestinal (GI) tract, and skin^7–12^. The concentration of αS is highly variable throughout the body ranging from low pM in samples such as saliva and skin, to significantly more concentrated in presynaptic terminals, where levels are reported to be in the region of 50–140 μM^13–16^. Therefore, suitable methods to detect αS, either in the brain or non-invasive peripheral fluids, and correlate this to disease onset and severity, is a highly important research aim in the PD field.

Many of the probes designed to monitor αS aggregation currently reported in the literature have a focus on detecting fibrils^17^. There are few examples that specifically detect αS oligomers and the earliest stages of αS aggregation^18,19^. However, developing probes specific to oligomers and conformers formed in the earliest stages of aggregation, would be most beneficial for developing an improved understanding of the disease pathway, in research for developing disease-modifying treatments, and in developing methods to diagnose the disease.

However, one of the main challenges facing the development of probes (and therapeutics) of αS aggregation is the vast array of protein–protein interactions (PPIs) that form throughout the aggregation pathway—from on- and off-pathway oligomers and conformers of a wide variety of sizes, through to amyloid fibrils^20^. PPIs are notoriously difficult to target with small molecules primarily due to the large, shallow target surface areas^21^. However, larger biological molecules—such as peptides—often provide much higher selectivity and affinity towards these interactions due to their ability to make many more contacts with the target. There are multiple possible mechanisms in which a peptide may be able to distinguish between different protein conformations, such as between different oligomers or aggregates. For example, the peptide may preferentially bind to a selective conformation of the protein (e.g. monomer vs. fibril), or by labelling a peptide with an environmentally-sensitive fluorophore, it may be possible to report on the range of binding environments of the peptide—for example, it may not selectively bind to monomer vs. oligomer, but the fluorescence response may differ in these two situations.

Herein we design and evaluate a peptide-based probe to monitor αS aggregation, with a particular focus to monitor the earliest stages of aggregation and the formation of oligomers. These probes could be developed for several potential applications including early diagnosis of PD (i.e. before symptoms begin), a method to evaluate the effectiveness of potential therapeutics, and as a tool to help in the understanding of the onset and development of PD. Many of the probes (small-molecule or peptide based) currently described in literature either are not specific to αS, or do not detect oligomers^17,22–24^. Therefore, herein the probes have been designed using a library derived peptide (4554W) previously identified by our group that is capable of binding to αS, that inhibits the earliest stages of aggregation, and impacts upon cytotoxicity^25^. Moreover, an analogue of the fluorophore 3-HF was chosen due to previous success in measuring Aβ aggregation^26^. Several methods have been used to help evaluate the success of the design of the probes including ThT assays to determine if the labelled-peptides are still effective at reducing aggregation of αS (if so, they have the potential to be used as theranostic); fluorescence polarization assays to determine if the probes can provide an alternative method of monitoring aggregation pathways, especially one that is more sensitive to the formation of oligomers; and an assay to determine if the spectra of the probe is sensitive to various early-oligomer samples.

## Results and discussion

### Peptide-probe design

Previously, an intracellular protein-fragment complementation assay (PCA) library screen (> 200,000 members) identified the 4554W peptide as an inhibitor of αS aggregation^25^. This semi-rational library was designed using 45–54 preNAC region of αS as the template due to all but one of the known mutations associated with early-onset PD being located within this region^27–31^. Subsequently, three further mutations have been identified in this region^32–36^, and recently published fibril structures have determined this region as being central to the formation of the fibril core and is therefore highly likely to be key in the misfolding of αS^37–39^. Herein, we use 4554W as the peptide backbone and modify it through labelling with an environmentally-sensitive fluorophore. The aim of this is to determine if this is a suitable probe for monitoring the aggregation and/or formation of oligomers as a potential method of PD diagnosis.

Previous work by Sedgwick et al.^26^ identified an excited-state intramolecular proton transfer (ESIPT) fluorophore based on 3-hydroxyflavone (3-HF) that resulted in an environmentally-sensitive response to the binding of different oligomers and aggregates of amyloid-β (Aβ). Here, we explore the potential to expand on this work to include the aggregation of αS by labelling our previously identified peptide binder (4554W) with a derivative of the 3-HF fluorophore.

**Figure 1:**
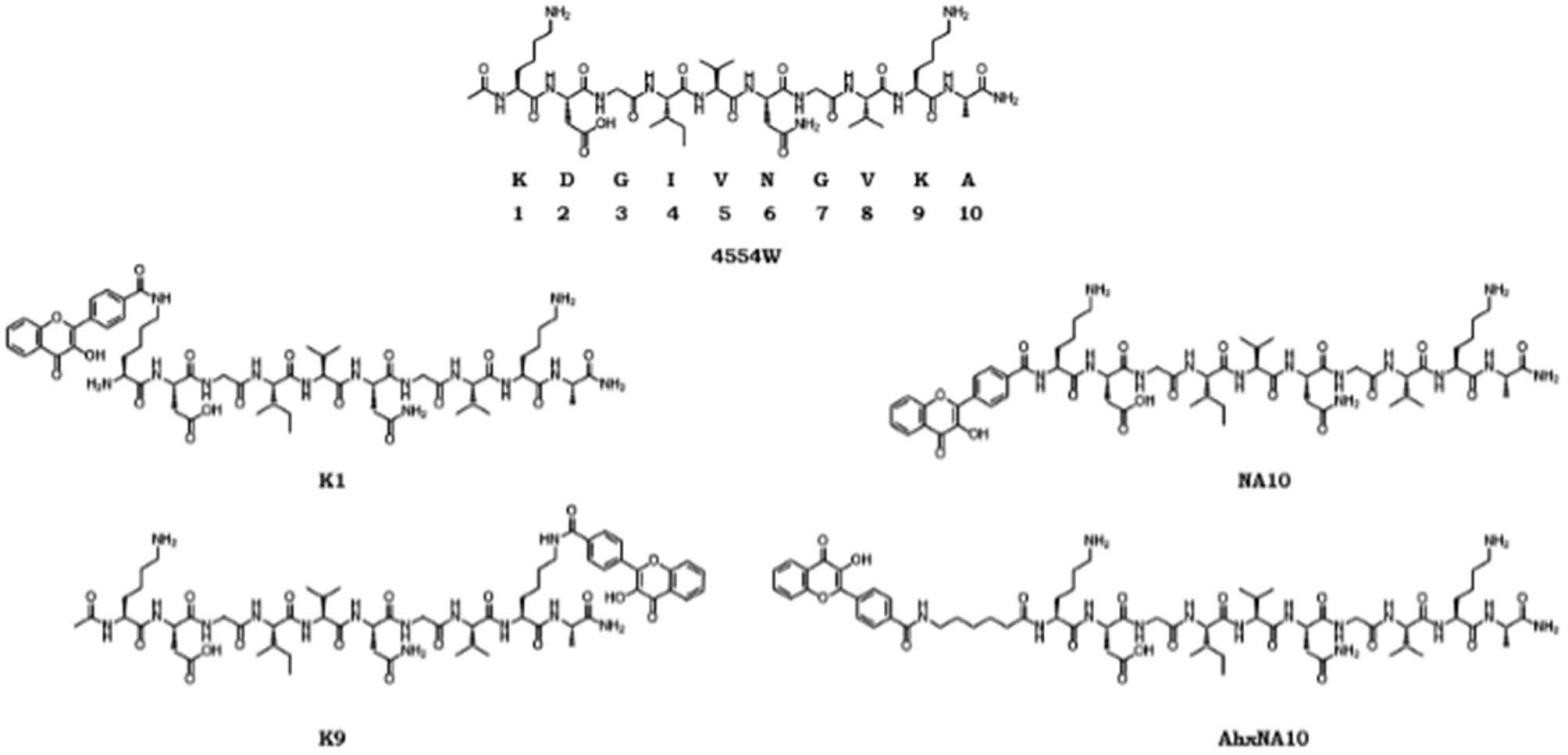
Summary of labelled-peptide structures. Original peptide structure (4554W), amino acid sequence and numbering, followed by the structure of the labelled peptides (K1, K9, NA10, and AhxNA10), showing the different positions of the fluorophore.

In order to assess if the position of the fluorophore affects the binding and/or fluorescence response, a series of labelled-peptides were designed and synthesised (Fig. 1). In addition to directly labelling the N-terminus (NA10), we also synthesised AhxNA10 that employs a 6-carbon linker to remove the fluorophore from the peptide backbone to prevent potential negative binding interactions that could arise from the steric bulk of the fluorophore. Additionally, we labelled the lysine positions (located at position 1 and 9) of the parent 4554W peptide resulting in the labelled-peptides K1 and K9. The lysine positions were chosen for multiple reasons including removal of the fluorophore from the peptide backbone, having two opposite points for comparison on the original structure, and ease of chemical synthesis. Additionally, we have previously shown that truncation of the peptide by removal of the lysine at the 1 position does not result in a loss of efficacy, therefore this residue does not appear necessary for binding, hence modification to it may not negatively affect binding^40^. Through a range of experiments described in the following sections, labelling of the 1 position (K1) proved to be the best-performing probe across all experiments.

### Peptide-probe performance: aggregation inhibition

**Figure 2:**
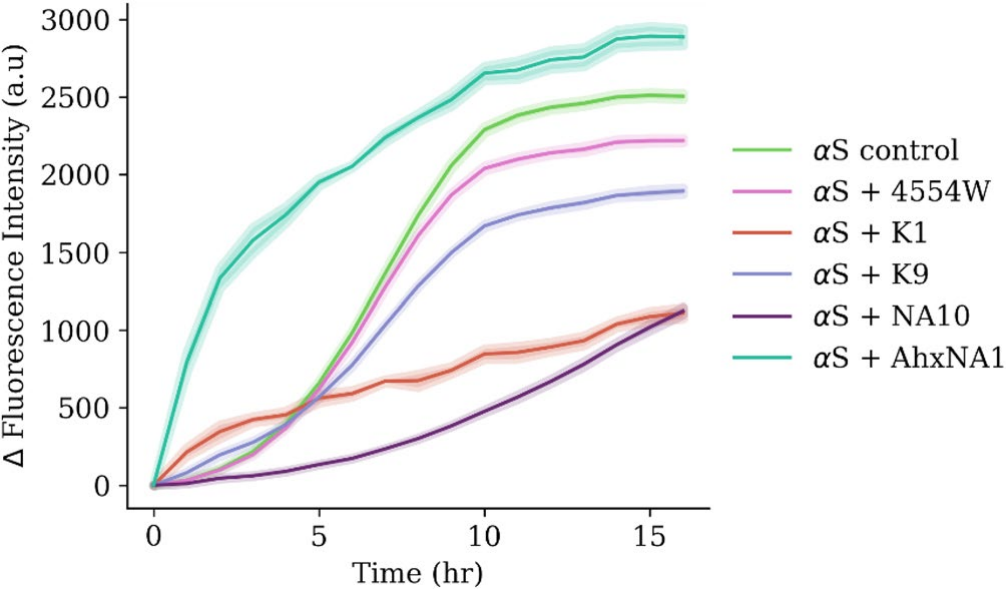
ThT aggregation of the labelled-peptides. Effect the labelled-peptides have on the inhibition of αS, using lipid-induced primary nucleation as monitored by ThT fluorescence (50 μM, λex 440 nm, λem 480 nm). αS (100 μM), DMPS SUVs (200 μM), labelled peptide (100 μM) and ThT (50 μM) in 20 mM sodium phosphate buffer (pH 6.5) were incubated at 30 °C under quiescent conditions for 16 h. The data shows the average of three repeats with the error bars representing the standard error.

Previous work has shown that the 4554W peptide is able to inhibit αS aggregation in a dose-dependent manner^25,41^. Therefore, we investigated if the labelled-peptides are able to maintain the inhibition potential of αS aggregation, and if so, these labelled-peptides could have potential to be developed as a diagnostic or theranostic, by both identifying aggregates and simultaneously sequestering them from further aggregation. Using the lipid-induced primary nucleation method previously determined to be inhibited by the parent peptide, 4554W, the effectiveness of the labelled-peptides was assessed. Monomeric αS (100 μM), labelled-peptide (100 μM, 1:1), DMPS SUVs (200 μM), and ThT (50 μM) in 20 mM PB, pH 6.5, were incubated under quiescent conditions (30 °C, 20 h) (for full methods, see Supplementary Information). DMPS lipid vesicle were chosen since they are a key component of dopaminergic synaptic vesicles, display a negatively charged headgroup, promote αS membrane binding, and lead to an increased local concentration of αS that accelerates primary nucleation^41^. αS aggregation was monitored using ThT which is excited with a wavelength of 440 nm, and emission measured at 480 nm. In contrast, the fluorophore used here has a maximum excitation of 365 nm and therefore does not interfere with the measurement of the ThT response to αS aggregation (i.e. FRET will not occur between the two fluorophores when monitoring ThT since the excitation and emission wavelengths are greater than that of the fluorophore excitation). Shown in Fig. 2, all but one (AhxNA10) of the labelled peptides resulted in a lower ThT intensity than the αS control, therefore these labelled-peptides enhance the inhibition of 4554W against αS aggregation. Whilst this was not a design feature in the development of an αS probe, it is favourable and indicative that peptide labelling does not result in loss of ability to interact with αS. Further, by maintaining the ability to inhibit aggregation, there is potential for these probes to be developed as theranostics.

### Peptide-probe performance: alternative to ThT to monitor aggregation

Thioflavin T (ThT) is the most widely used method to monitor the aggregation of αS and other amyloid proteins in in vitro assays^42^. It works through an increase in the emission fluorescence intensity (FI) when it is bound to amyloid fibrils that are rich in β-sheets, however, this means that it often cannot be used to detect pre-fibrillar species, like those assumed to be the ones of most interest within amyloid diseases, such as PD^41,43^. Furthermore, the accuracy of an FI measurement depends on the homogeneity of the solution. In solutions containing aggregated protein, the solution is not homogeneous: the density of aggregates throughout the sample will vary. One method to overcome this is to measure fluorescence polarization (FP). FP is a technique that measures the amount of polarized light, in contrast to the intensity of the fluorescence as in ThT assays. This helps to overcome some limitations, such as amyloid aggregates blocking some of the fluorescence signal resulting in a lower signal than is true. FP is based on the principle that the degree of polarization emitted when a fluorescent probe is excited with polarized light, is inversely proportional to the rate of molecular rotation^44^. Therefore, if a fluorophore is bound to a protein as it aggregates, then it would be expected that the extent of polarization will increase with time. Therefore, the suitability of the labelled-peptide probes to be used as a potential tool in monitoring the aggregation of αS was investigated herein.

**Figure 3:**
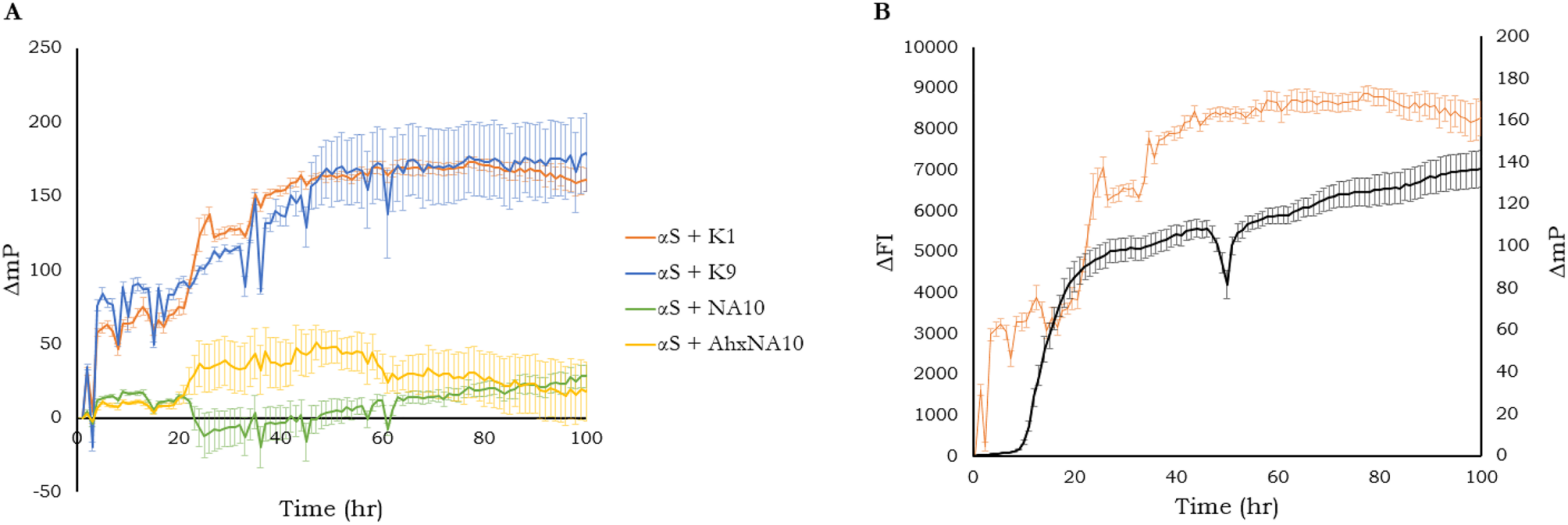
Measuring the suitability of labelled-peptides to monitor αS aggregation through fluorescence polarization (FP). (**A**) FP measurement of the four labelled-peptides (10 μM; K1 (orange), K9 (blue), NA10 (green), and AhxNA10 (yellow)) incubated with αS (200 μM) under aggregating conditions (37 °C, 700 rpm double orbital shaking, 100 h). (**B**) Comparison between ThT and K1 to monitor αS aggregation. Black line represents the change in fluorescence intensity of ThT (150 µM, λex = 450 nm, λem = 482 nm), the orange line represents the change in fluorescence polarization (mP) of probe K1. Both samples are incubated with αS (200 μM) and K1(10 μM). At this 20 × excess of aS:peptide no inhibitory activity was previously shown for 4554W^39^. Assay conditions: 37 °C, continuous shaking at 700 rpm double orbital shaking, 100 h; polarizing filters: ex = 360 nm, em = 530 nm. Data shown represent a single experiment with three technical replicates.

To determine if the labelled-peptides are capable of monitoring the progression of αS aggregation as an alternative to ThT, the labelled-peptides (10 μM) and αS (200 μM) were incubated under harsh aggregation conditions (shaking at 700 rpm, 37 °C, 100 h), and the change in polarization with time for each of the labelled-peptides was plotted (Fig. 3A) (for full methods, see Supplementary Information). From these results, it was clear that K1 and K9 are much better suited for monitoring the aggregation pathway of αS than NA10 and AhxNA10, due to both K1 and K9 resulting in significant increase in polarization with time. In order to verify that the results from Fig. 3A correspond to the aggregation of αS, the αS aggregation profile monitored by K1 (change in FP (∆mP)) was compared to ThT (change in fluorescence intensity (∆FI)) (Fig. 3B). In Fig. 3B, the increase in polarization of K1 (orange) precedes that of the fluorescence intensity of ThT (black), indicating that K1 may be more sensitive to pre-fibril species than ThT. These results suggest that these probes—in particular K1—could be a suitable alternative to ThT as a method to monitor the aggregation pathway of αS. Monitoring the earliest stages of aggregation is a particular downfall of ThT as the species formed in this stage are assumed to be of most interest with regards to developing diagnostic and therapeutic strategies. Therefore, K1 has potential to evaluate the ability of novel therapeutics to modulate early events within the aggregation process.

### Peptide-probe performance: fluorophore response to αS oligomers

Development of a method to easily and reliably measure the oligomeric content of an αS sample would have great benefits within the PD research field. For example, developing a probe that could specifically detect biologically or disease-relevant αS oligomers in the brain or peripheral fluids could be hugely beneficial in the development of an improved early method of diagnosis. Furthermore, such a probe could help to evaluate the potential of novel therapeutics in their ability to modulate the formation and toxicity of oligomers. Throughout the aggregation pathway, a wide variety of structures form that, for example, vary in the extent of β-sheet structure or the exposure of hydrophobic resides^45^. Therefore, the aim of this section was to determine if the labelled-peptides are sensitive to the environmental changes resulting from various αS species, and consequently if the labelled-peptides are capable of producing characteristic signals with different types of oligomeric structures.

**Figure 4:**
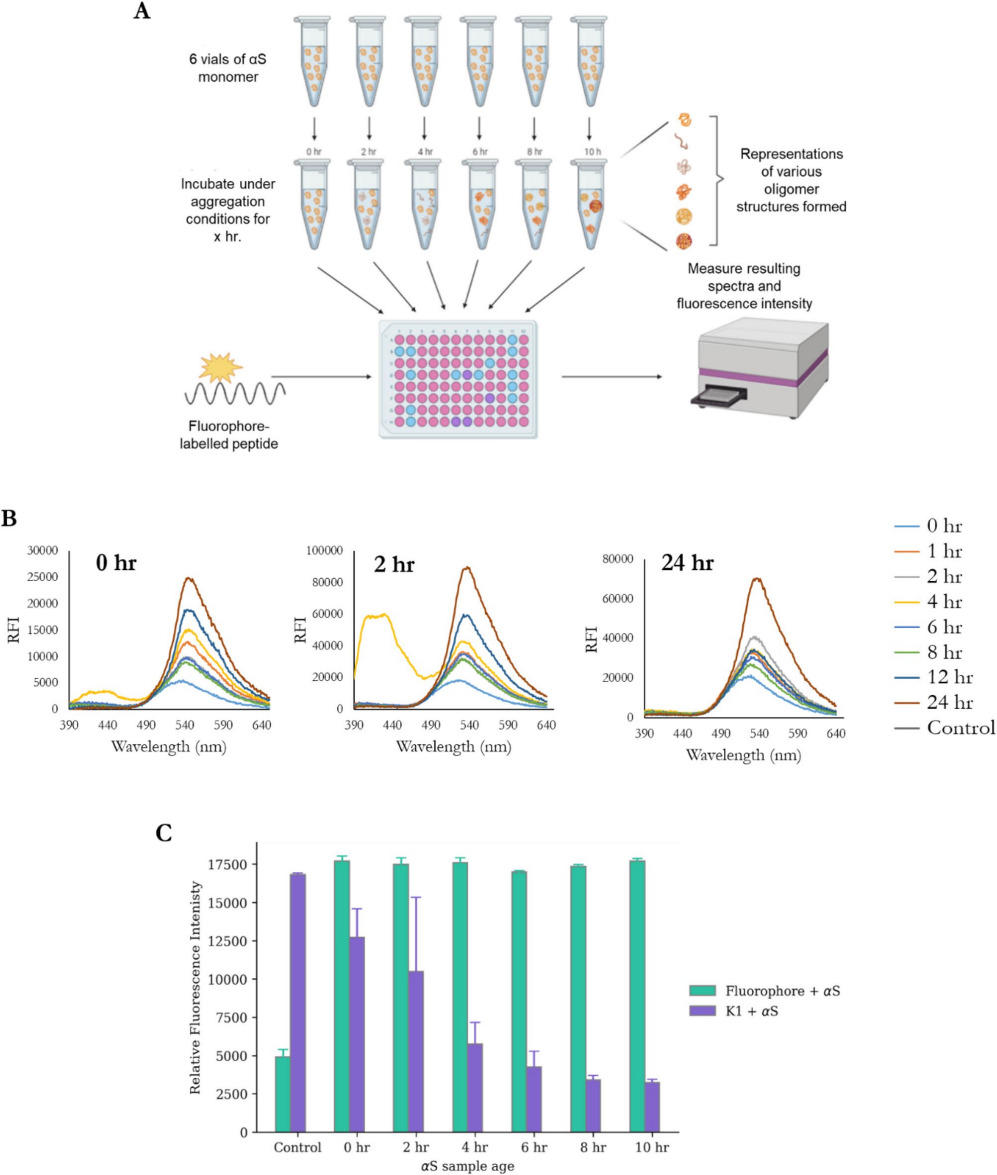
“Aged”-αS experiment design and results. (**A**) Schematic describing αS aging method. Identical vials of αS monomer (500 μM) were incubated under aggregating conditions (700 rpm double orbital shaking, 37 °C) for a set period of time. It is assumed that during these varying lengths of aggregation that a range of oligomeric species will form. Aged αS samples were added to a 96-well microplate along with the labelled-peptides, and the resulting fluorescence spectra were obtained on a CLARIOstar plate reader. Figure made in BioRender.com. (**B**) On a single occasion (n = 1), the N peak was observed when K1 (25 μM) was added to a sample of αS (50 μM) aged for the specified length of time (0, 1, 2, 4, 6, 8, 12, or 24 h) and the spectra (λex 360 nm, λem 390–650 nm) was recoded at 0 h, after 2 h incubation, and after 24 h incubation. (**C**) To the aged αS sample (50 μM), either the fluorophore (25 μM) or K1 (25 μM) was added and the resulting spectra (λex = 360 nm, λem 390–650 nm) was recorded. Plotting the intensity of the T peak (540 nm) for each of the samples showed that the intensity of the fluorophore alone did not vary depending on the age of the αS sample, whereas there was a strong decreasing trend in intensity correlating to αS sample age with K1. The results are the average of three repeats, with the error bars representing standard error.

In order to determine if it is possible to take advantage of the environmentally-sensitive ESIPT fluorescence of these labelled-peptides for the detection of αS oligomers, an assay was designed. Monomeric αS (500 μM) was “aged” through incubation under aggregating conditions (700 rpm double orbital shaking, 37 °C) for up to 24 h (Fig. 4A) (for full methods, see Supplementary Information). This relatively short period of time has previously been shown to corresponded to the lag-phase of αS aggregation, therefore various oligomers or other non-ThT-active pre-fibrillar species are expected to form. To these aged αS samples (50 μM), the labelled-peptides (25 μM) were added and the resulting spectra were measured (Fig. 4B). Based on typical behaviour of 3-HF fluorophores, a number of possible spectral changes were expected, for example we observed in increasing T peak intensity with increasing hydration, and an increase in N peak intensity with decreased polarity.

The labelled-peptide that exhibited the largest variety of response to the aged αS samples was K1, where all of the samples had varying intensities of the T (λ_MAX_ 540 nm) peak. Notably, on one occasion, the 4 h sample had a small distinct N (λ_MAX_ 420 nm) peak (Fig. 4B). Following a short incubation (2 h, 37 °C), the spectra were re-recoded. At this point, a very distinct N peak for K1 was observed (4 h αS). Furthermore, following a longer incubation (24 h, 37 °C), the spectra were re-recoded again, and, in this case, the N peak from the K1 sample was not observed. These results appear to indicate that the probes are responsive to environmental changes/ differing αS species, and that the oligomer populations formed continue to be dynamic during incubation. Moreover, the results indicated that the N peak may be incredibly sensitive to a particular binding environment. However, whilst initially promising, further work to expand on the initial results highlighted that monitoring the N peak as a measurement of environmental response with this particular fluorophore may be challenging. Whilst there does appear to be a response to different samples, there appeared to be no predicable correlation as to when this specific response of increasing intensity of the N peak would occur.

More promisingly however was monitoring the intensity of the T peak, in particular for labelled-peptide K1. In Fig. 4C, the maximum intensity of the T peak (535 nm) of the fluorophore alone and for labelled-peptide K1 when incubated with the aged αS samples (following 2 h equilibration) is compared. Two trends were observed from this data—first being that the intensity of K1 T peak decreases markedly with the αS sample age, and second, that the fluorophore alone (not conjugated to the peptide) intensity does not vary with αS sample age, indicating that conjugation to the peptide is essential. This suggests that the peptide is required for specificity, and this may result in an αS-specific probe. Of note, in this set of experiments, in contrast to the spectra shown in B, no N peak was observed.

## Conclusions

Despite the prevalence of PD, there are currently no disease modifying treatments available, although new approaches are beginning to emerge that includes amplifying the amount of misfolded αS to enable detection and diagnsosis^46^. One significant reason for the lack of suitable treatments, has been such effective early diagnose of the disease (i.e. where disease-modifying treatments would be most effective), and the inability to monitor disease progression at a biochemical level^47,48^. Towards addressing this challenge, herein we designed and evaluated several fluorophore-labelled peptide probes with the aim to ultimately develop this as a method of early diagnosis, or as a tool to monitor disease progression, through their ability to identify oligomeric species of αS.

Labelling the 4554W peptide at position K1 with an environmentally-sensitive fluorophore was found to result in the most effective probe across all experiments. This probe was found to be an enhanced inhibitor of αS aggregation compared to the parent peptide (4554W), and was more sensitive to pre-fibrillar species in the αS aggregation pathway than the widely used fluorescent probe ThT. Moreover, the peptide was found to produce a differential response in the presence of various αS oligomer samples, indicating that it may be useful as an αS-oligomer probe, with further work required to increase sensitivity of detection. Overall, this work has determined that that fluorophore-labelled 4554W (K1) has significant potential for further development and optimisation as an αS oligomer sensitive probe for a number of applications within PD research.

## Supplementary Information

Supplementary Information.

## Data Availability

The datasets used and/or analysed during the current study are available from the corresponding author on reasonable request.

## References

[1] Kalia LV, Lang AE (2015). Parkinson's disease. Lancet.

[2] Miller DB, O’Callaghan JP (2015). Biomarkers of Parkinson’s disease: Present and future. Metab. Clin. Exp..

[3] Karaboğa MNS, Sezgintürk MK (2022). Biosensor approaches on the diagnosis of neurodegenerative diseases: Sensing the past to the future. J. Pharm. Biomed..

[4] Lansbury PT (2004). Back to the future: The 'old-fashioned' way to new medications for neurodegeneration. Nat. Med..

[5] Fayyad M, Salim S, Majbour N, Erskine D, Stoops E, Mollenhauer B, El-Agnaf OMA (2019). Parkinson's disease biomarkers based on α-synuclein. J. Neurochem..

[6] Wang J, Hoekstra JG, Zuo C, Cook TJ, Zhang J (2013). Biomarkers of Parkinson's disease: Current status and future perspectives. Drug Discov. Today.

[7] Atik A, Stewart T, Zhang J (2016). Alpha-synuclein as a biomarker for Parkinson's Disease. Brain Pathol..

[8] Borghia R, Marcheseb R, Negroc A, Marinellia L, Forlonid G, Zaccheoa D, Abbruzzeseb G, Tabaton M (2000). Full length alpha-synuclein is present in cerebrospinal fluid from Parkinson's disease and normal subjects. Neurosci. Lett..

[9] Tokuda T, Salem SA, Allsop D, Mizuno T, Nakagawa M, Qureshi MM, Locascio JJ, Schlossmacher MG, El-Agnaf OMA (2006). Decreased alpha-synuclein in cerebrospinal fluid of aged individuals and subjects with Parkinson's disease. Biochem. Biophys. Res. Commun..

[10] Gelpi E, Navarro-Otano J, Tolosa E, Gaig C, Compta Y, Rey MJ, Martí MJ, Hernández I, Valldeoriola F, Reñé R, Ribalta T (2014). Multiple organ involvement by alpha-synuclein pathology in Lewy body disorders. Mov. Disord..

[11] Fricova D, Harsanyiova J, Kralova Trancikova A (2020). Alpha-synuclein in the gastrointestinal tract as a potential biomarker for early detection of Parkinson’s Disease. Int. J. Mol. Sci..

[12] Doppler K (2021). Detection of dermal alpha-synuclein deposits as a biomarker for Parkinson’s Disease. J. Park. Dis..

[13] van Raaij ME, van Gestel J, Segers-Nolten IMJ, de Leeuw SW, Subramaniam V (2008). Concentration dependence of alpha-synuclein fibril length assessed by quantitative atomic force microscopy and statistical-mechanical theory. Biophys. J..

[14] Wilhelm BG, Mandad S, Truckenbrodt S, Kröhnert K, Schäfer C, Rammner B, Koo SJ, Claßen GA, Krauss M, Haucke V, Urlaub H, Rizzoli SO (2014). Composition of isolated synaptic boutons reveals the amounts of vesicle trafficking proteins. Science.

[15] Vivacqua G, Suppa A, Mancinelli R, Belvisi D, Fabbrini A, Costanzo M, Formica A, Onori P, Fabbrini G, Berardelli A (2019). Salivary alpha-synuclein in the diagnosis of Parkinson's disease and Progressive Supranuclear Palsy. Park. Relat. Disord..

[16] Chang C-W, Yang S-Y, Yang C-C, Chang C-W, Wu Y-R (2020). Plasma and serum alpha-synuclein as a biomarker of diagnosis in patients with parkinson's disease. Front Neurol.

[17] Xu M-M, Ryan P, Rudrawar S, Quinn RJ, Zhang H-Y, Mellick GD (2020). Advances in the development of imaging probes and aggregation inhibitors for alpha-synuclein. Acta Pharmacol. Sin..

[18] Wang R (2023). A review of the current research on in vivo and in vitro detection for alpha-synuclein: A biomarker of Parkinson's disease. Anal. Bioanal. Chem..

[19] Haque R, Maity D (2023). Small molecule-based fluorescent probes for the detection of alpha-Synuclein aggregation states. Bioorg. Med. Chem. Lett..

[20] Meade RM, Fairlie DP, Mason JM (2019). Alpha-synuclein structure and Parkinson's disease—Lessons and emerging principles. Mol. Neurodegener..

[21] Ran X, Gestwicki JE (2018). Inhibitors of protein-protein interactions (PPIs): An analysis of scaffold choices and buried surface area. Curr. Opin. Chem. Biol..

[22] Lee JH, Lee IH, Choe YJ, Kang S, Kim HY, Gai WP, Hahn JS, Paik SR (2009). Real-time analysis of amyloid fibril formation of α-synuclein using a fibrillation-state-specific fluorescent probe of JC-1. Biochem. J..

[23] Leung CWT, Guo F, Hong Y, Zhao E, Kwok RTK, Lik N, Leung C, Chen S, Vaikath NN, El-Agnaf OM, Tang Y, Gai W-P, Tang BZ (2015). Detection of oligomers and fibrils of α-synuclein by AIEgen with strong fluorescence. Chem. Commun..

[24] Wood A, Chau E, Yang Y, Kim JR (2020). A KLVFFAE-derived peptide probe for detection of alpha synuclein fibrils. Appl. Biochem. Biotechnol..

[25] Cheruvara H, Allen-Baume VL, Kad NM, Mason JM (2015). Intracellular screening of a peptide library to derive a potent peptide inhibitor of α-synuclein aggregation. J. Biol. Chem..

[26] Sedgwick AC, Dou W-T, Jiao J-B, Wu L, Williams GT, Jenkins ATA, Bull SD, Sessler JL, He X-P, James TD (2018). An ESIPT probe for the ratiometric imaging of peroxynitrite facilitated by binding to Aβ-aggregates. J. Am. Chem. Soc..

[27] Polymeropoulos MH, Lavedan C, Leroy E, Ide SE, Dehejia A, Dutra A, Pike B, Root H, Rubenstein J, Boyer R, Stenroos ES, Chandrasekharappa S, Athanassiadou A, Papapetropoulos T, Johnson WG, Lazzarini AM, Duvoisin RC, Di Iorio G, Golbe LI, Nussbaum RL (1997). Mutation in the alpha-synuclein gene identified in families with Parkinson's disease. Science.

[28] Krüger R, Kuhn W, Müller T, Woitalla D, Graeber M, Kösel S, Przuntek H, Epplen JT, Schöls L, Riess O (1998). Ala30Pro mutation in the gene encoding alpha-synuclein in Parkinson's disease. Nat. Genet..

[29] Zarranz JJ, Alegre J, Gómez-Esteban JC, Lezcano E, Ros R, Ampuero I, Vidal L, Hoenicka J, Rodriguez O, Atarés B, Llorens V, Tortosa EG, del Ser T, Muñoz DG, de Yebenes JG (2004). The new mutation, E46K, of alpha-synuclein causes Parkinson and Lewy body dementia. Ann. Neurol..

[30] Appel-Cresswell S, Vilarino-Guell C, Encarnacion M, Sherman H, Yu I, Shah B, Weir D, Thompson C, Szu-Tu C, Trinh J, Aasly JO, Rajput A, Rajput AH, Stoessl AJ, Farrer MJ (2013). Alpha-synuclein p.H50Q, a novel pathogenic mutation for Parkinson's disease. Mov. Disord..

[31] Proukakis C, Dudzik CG, Brier T, Mackay DS, Cooper JM, Millhauser GL, Houlden H, Schapira AH (2013). A novel α-synuclein missense mutation in Parkinson disease. Neurology.

[32] Kiely AP, Asi YT, Kara E, Limousin P, Ling H, Lewis P, Proukakis C, Quinn N, Lees AJ, Hardy J, Revesz T, Houlden H, Holton JL (2013). α-Synucleinopathy associated with G51D SNCA mutation: A link between Parkinson's disease and multiple system atrophy?. Acta Neuropathol..

[33] Pasanen P, Myllykangas L, Siitonen M, Raunio A, Kaakkola S, Lyytinen J, Tienari PJ, Pöyhönen M, Paetau A (2014). A novel α-synuclein mutation A53E associated with atypical multiple system atrophy and Parkinson's disease-type pathology. Neurobiol. Aging.

[34] Martikainen MH, Päivärinta M, Hietala M, Kaasinen V (2015). Clinical and imaging findings in Parkinson disease associated with the A53E SNCA mutation. Neurol. Genet.

[35] Yoshino H, Hirano M, Stoessl AJ, Imamichi Y, Ikeda A, Li Y, Funayama M, Yamada I, Nakamura Y, Sossi V, Farrer MJ, Nishioka K, Hattori N (2017). Homozygous alpha-synuclein p.A53V in familial Parkinson's disease. Neurobiol. Aging.

[36] Lesage S, Anheim M, Letournel F, Bousset L, Honoré A, Rozas N, Pieri L, Madiona K, Dürr A, Melki R, Verny C, Brice A (2013). G51D α-synuclein mutation causes a novel parkinsonian-pyramidal syndrome. Ann. Neurol..

[37] Li B, Ge P, Murray KA, Sheth P, Zhang M, Nair G, Sawaya MR, Shin WS, Boyer DR, Ye S, Eisenberg DS, Hong Zhou Z, Jiang L (2018). Cryo-EM of full-length α-synuclein reveals fibril polymorphs with a common structural kernel. Nat. Commun..

[38] Guerrero-Ferreira R, Taylor NMI, Mona D, Ringler P, Lauer ME, Riek R, Britschgi M, Stahlberg H (2018). Cryo-EM structure of alpha-synuclein fibrils. Elife.

[39] Tuttle MD, Comellas G, Nieuwkoop AJ, Covell DJ, Berthold DA, Kloepper KD, Courtney JM, Kim JK, Barclay AM, Kendall A, Wan W, Stubbs G, Schwieters CD, Lee VMY, George JM, Rienstra CM (2016). Solid-state NMR structure of a pathogenic fibril of full-length human α-synuclein. Nat. Struct. Mol. Biol..

[40] Meade RM, Watt KJC, Williams RJ, Mason JM (2021). A downsized and optimised intracellular library-derived peptide prevents alpha-synuclein primary nucleation and toxicity without impacting upon lipid binding. J. Mol. Biol..

[41] Meade RM, Morris KJ, Watt KJC, Williams RJ, Mason JM (2020). The library derived 4554W peptide inhibits primary nucleation of α-synuclein. J. Mol. Biol..

[42] Biancalana M, Koide S (2010). Molecular mechanism of Thioflavin-T binding to amyloid fibrils. Biochim. Biophys. Acta.

[43] Needham L-M, Weber J, Varela JA, Fyfe JWB, Do DT, Xu CK, Tutton L, Cliffe R, Keenlyside B, Klenerman D, Dobson CM, Hunter CA, Müller KH, Bohndiek SE, Snaddon TN, Lee SF (2020). ThX—A next-generation probe for the early detection of amyloid aggregates. Chem. Sci..

[44] Moerke NJ (2009). Fluorescence polarization (FP) assays for monitoring peptide-protein or nucleic acid-protein binding. Curr. Protoc. Chem. Biol..

[45] Roberts HL, Brown DR (2015). Seeking a mechanism for the toxicity of oligomeric α-synuclein. Biomolecules.

[46] Siderowf A (2023). Assessment of heterogeneity among participants in the Parkinson's Progression Markers Initiative cohort using alpha-synuclein seed amplification: A cross-sectional study. Lancet Neurol..

[47] Gadhe L (2022). Intermediates of alpha-synuclein aggregation: Implications in Parkinson's disease pathogenesis. Biophys. Chem..

[48] Nguyen PH (2021). Amyloid oligomers: A joint experimental/computational perspective on Alzheimer's disease, Parkinson's disease, type II diabetes, and amyotrophic lateral sclerosis. Chem. Rev..

