# Supplementary material for: Development of a hydroxyflavone-labelled 4554W peptide probe for monitoring αS aggregation
Source: Sci Rep. 2023 Jul 6;13:10968. doi: 10.1038/s41598-023-37655-3 (PMC10326036; doi:10.1038/s41598-023-37655-3)
Supplement: Supplementary file 1 — Supplementary Information. [file 41598_2023_37655_MOESM1_ESM.docx]

Supporting Information

**Development of a hydroxyflavone-labelled 4554W peptide probe for αS aggregation**

Kathryn J.C. Watt, Richard M. Meade, Tony D. James and Jody M. Mason

## **Fluorophore synthesis**

General overview:


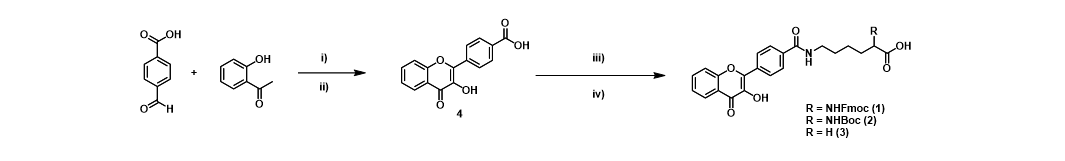


**Figure S1:** **General overview of synthesis for fluorophore-labelled amino acids.** Synthesis is as follows: **i)** NaOH, MeOH, ∆, 2 h; **ii)** H_2_O_2_, NaOH (0.5 N), RT, 3 h; **iii)** NHS, EDC.HCl, DMF, RT, o/n; **iv)** Fmoc-Lys-OH (**1**), Boc-Lys-OH (**2**), or Ahx-OH (**3**), RT, o/n.


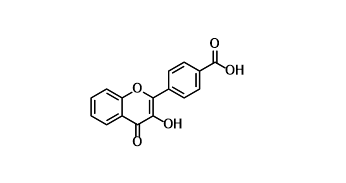
*Synthesis of 4-(3-hydroxy-4-oxo-4H-chromen-2-yl)benzoic acid* (**4**)^283^

4-formylbenzoic acid (1.46 g, 9.72 mmol), 2’-hydroxyphenone (1.2 mL, 10 mmol), and NaOH (1.2 g, 30 mmol) were dissolved in MeOH (60 mL) and heated to reflux overnight. The solution was cooled to RT before NaOH (0.5 N, 60 mL) and H_2_O_2_ (30%, 4.8 mL) were added. The resulting solution was stirred at RT for 3 h before being poured into ice-water and acidified with HCl (1 M). The resulting precipitate was filtered and washed with MeOH, resulting in a pale yellow solid (**4**). (1.16 g, 42%). The product was used without any further purification. **^1^H NMR** (300 MHz, DMSO-d_6_) δ_H_: 13.21 (1H, s), 10.00 (1H, s), 8.36-8.32 (2H, m), 8.13-8.07 (3H, m), 7.85-7.76 (2H, m), 7.50-7.44 (1H, m).

*Synthesis of N2-(((9H-fluoren-9-yl)methoxy)carbonyl)-N6-(4-(3-hydroxy-4-oxo-4H-chromen-2-yl)benzoyl)lysine* (**1**)
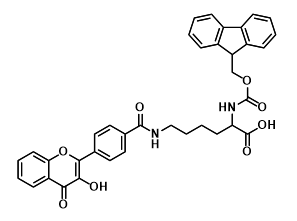


EDC.HCl (1.01 g, 5.27 mmol) was added to a cooled solution (0 °C) of 4-(3-hydroxy-4-oxo-4H-chromen-2-yl)benzoic acid (**4**) (990 mg, 3.51 mmol) and N-hydroxysuccinimide (605 mg, 5.26 mmol) in DMF (50 mL). The resulting solution was stirred at 0 °C for 20 minutes before stirring at RT for 24 h. Fmoc-Lys-OH (2.01 g, 5.46 mmol) was added and the resulting solution was stirred at RT for 3 days. The yellow suspension was filtered and the resulting filtrate was extracted with ethyl acetate and water. The organic layer was dried over MgSO_4_, filtered and concentrated under reduced pressure. The resulting residue was triturated in toluene, followed by diethyl ether. The suspension was filtered, resulting in the desired product (**1**) as a pale yellow solid (455 mg, 21%). The product was used without any further purification. **^1^H NMR** (300 MHz, DMSO-d_6_) δ_H_: 8.63 (1H, s), 8.29 (2H, d, *J* 8.2 Hz), 8.12 (1H, d, *J* 7.9 Hz), 7.99 (2H, d, *J* 8.5 Hz), 7.86 (3H, d, *J* 7.4 Hz), 7.81-7.76 (1H, m), 7.70 (3H, d, *J* 7.4 Hz), 7.51 –7.43 (2H, m), 7.41-7.26 (6H, m), 4.34 – 4.13 (4H, m), 3.91 (1H, s), 1.46 (6H, m), 1.07(2H, m); **^13^C NMR** (75 MHz, DMSO-d_6_) δ_C_: 174.41, 173.46, 165.78, 156.47, 154.93, 144.50, 144.14, 141.03, 140.07, 135.63, 134.28, 133.97, 127.97, 127.71, 127.64, 127.40, 125.63, 125.17, 121.60, 120.45, 118.86, 79.52, 65.91, 54.21, 46.97, 30.92, 29.06, 23.54; **MS (ESI)** Calc. for C_37_H_32_N_2_O_8_Na [M+Na]^+^ 655.6483, found 655.2047


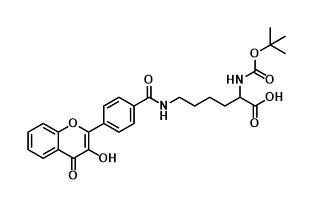
*Synthesis of N2-(tert-butoxycarbonyl)-N6-(4-(3-hydroxy-4-oxo-4H-chromen-2-yl)benzoyl)lysine* (**2**)

EDC.HCl (917 mg, 4.78 mmol) was added to a cooled solution (0 °C) of 4-(3-hydroxy-4-oxo-4H-chromen-2-yl)benzoic acid (**4**) (900 g, 3.19 mmol) and N-hydroxysuccinimide (550 mg, 4.78 mmol) in DMF (40 mL). The resulting solution was stirred at 0 °C for 20 minutes before stirring at RT overnight. Boc-Lys-OH (1.25 g, 5.08 mmol) was added and the resulting solution was stirred at RT overnight. The solution was extracted with ethyl acetate and water, and the organic layer was dried over MgSO_4_, filtered and concentrated under reduced pressure. The resulting residue was triturated in toluene followed by chloroform and diethyl ether. The resulting pale peach solid (**2**) was obtained by filtration (226 mg, 14%). The product was used without any further purification. **^1^H NMR** (300 MHz, DMSO-d_6_) δ_H_: 12.44 (1H, s), 9.89 (1H, s), 8.59(1H, dt, *J* 16.4, 5.6 Hz), 8.35 – 8.25 (2H, m), 8.16 – 8.07 (1H, m), 7.99 (2H, d, *J* 8.4Hz), 7.93 – 7.75 (2H, m), 7.47 (1H, ddd, *J* 8.1, 5.9, 2.2 Hz), 7.06 (1H, dd, *J* 8.0, 4.7Hz), 3.93 – 3.70 (2H, m), 3.26 (3H, d, *J* 7.2 Hz), 1.72 – 1.43 (4H, m), 1.37 – 1.31 (9 H, s); **^13^C NMR** (75 MHz, DMSO-d_6_) δ_C_: 174.66, 173.45, 165.78, 155.96, 154.95, 144.52, 140.05, 137.04, 134.31, 133.98, 127.72, 127.64, 127.41, 125.19, 125.04, 121.60, 118.88, 78.29, 53.77, 30.78, 29.05, 28.54, 23.50; **MS (ESI)** Calc. for C_27_H_30_N_2_O_8_Na [M+Na]^+^ 533.1894, found 533.1909.


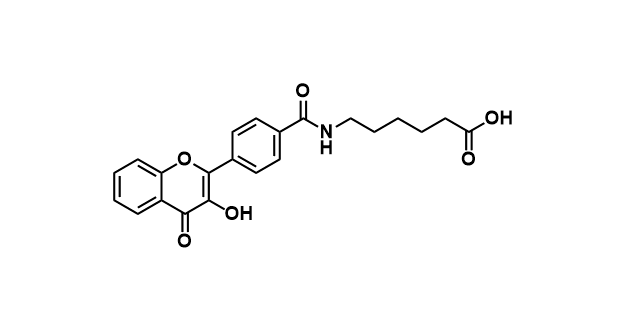
*Synthesis of 6-(4-(3-hydroxy-4-oxo-4H-chromen-2-yl)benzamido)hexanoic acid* (**3**)

EDC.HCl (1.08 g, 5.63 mmol) was added to a cooled solution (0 °C) of 4-(3-hydroxy-4-oxo-4H-chromen-2-yl)benzoic acid (**4**) (1.10 g, 3.90 mmol) and N-hydroxysuccinimide (621 mg, 5.40 mmol) in DMF (50 mL). The resulting solution was stirred at 0 °C for 30 minutes before stirring at RT o/n. 6-aminocaproic acid (1.02 g, 7.78 mmol) was added and the resulting solution was stirred at RT for 3 days. The solution was extracted with ethyl acetate and water, and the organic layer was dried over MgSO_4_, filtered and concentrated under reduced pressure, resulting in the desired product (**3**) as a pale yellow solid (1.12 g, 70%). The product was used without any further purification. **^1^H NMR** (300 MHz, DMSO-d_6_) δ_H_: 12.01 (1H, s), 9.89 (1H, s), 8.61 (1H, t, *J* 5.6Hz), 8.34 – 8.26 (2H, d, *J* 8.3 Hz), 8.12 (1H, d, *J* 7.9 Hz), 7.99 (2H, d, *J* 8.3 Hz), 7.86 – 7.76 (2H, m), 7.47 (1H, ddd, *J* 8.0, 5.8, 2.3 Hz), 3.29 – 3.23 (2H, m), 2.19 (2H, m), 1.53-1.48 (4H, m), 1.39-1.25 (2H, m); **^13^C NMR** (75 MHz, DMSO-d_6_) 174.84, 173.45, 165.75, 154.96, 144.54, 140.05, 135.68, 134.29, 133.97, 127.73, 127.63, 125.19,125.02, 121.62, 118.87, 36.14, 33.96, 29.22, 26.41, 24.62; **MS(ESI):** Calc for C_22_H_21_NO_6_ [M+H]^+^ 396.4123, found 396.1438

## **Peptide synthesis**

Peptides were synthesised on a H-Rink Amide Chem matrix resin (0.22 g, 0.1 mmol) using a Liberty Blue microwave peptide synthesiser (CEM) and standard techniques of Fmoc solid-phase peptide synthesis (SPPS).

Coupling of Fmoc-protected amino acids was achieved via double-coupling using Fmoc-protected amino acid (1 mmol), (Benzotriazol-1-yloxy)tripyrrolidinophosphonium hexafluorophosphate (PyBOP)(0.5 M in DMF) and N,N-Diisopropylethylamine (DIPEA)(17% in DMF (v/v)). Following coupling, piperidine (20% in DMF, with 5% formic acid) was used to deprotect the Fmoc-protecting group. 5% formic acid was required in order to minimises aspartimide formation.^262^

Following synthesis, the peptides were cleaved from the resin with simultaneous removal of the side-chain protecting groups through addition of a cleavage solution (10 mL: TFA (95%), triisopropylsilane (2.5%) and H_2_O (2.5%)) for 3.5 hours at RT. The cleaved resin was removed by filtration, and the filtrate was precipitated into ice-cold diethyl ether. The peptide pellet was obtained via vortexing, followed by centrifugation (7000 rcf, 10 minutes, 4 °C). This method was repeated three times with ice-cold diethyl ether added to the crude peptide pellet.

The resulting crude peptide was dissolved in HPLC buffer (5% ACN, 95% H_2_O). Purification was performed using a preparative scale reverse phase HPLC using a Phenomenex Jupiter Proteo reverse-phase column (4 μm, 90 Å, 250 x 21.2 mm), using eluents A (H_2_O with 0.1% TFA) and B (ACN with 0.1% TFA). Collected fractions were analysed by electrospray mass spectrometry (ESI). Those found to contain the desired product were pooled and lyophilized. The purified pellet was stored at -80 °C.

## **αS expression and purification**

Full-length (1-140) α-synuclein (αS) was recombinantly expressed in BL21(DE3) *E. coli* cells using a pET21a plasmid (WT αS plasmid was a gift from the Michael J. Fox Foundation, Addgene plasmid # 51486) and purified based on previously published methods.

Briefly, overnight cultures (2XYT, 10 mL) of the transformed *E. coli* were used to inoculate 2XYT (1 L) cultures containing ampicillin (100 mgL^-1^) which were grown (37 °C, 200 rpm) to an OD_600_ of 0.6-0.8. Protein expression was induced with IPTG (final concentration of 1 mM) and cells were harvested by centrifugation (5000 rpm, 20 min, 4 °C) following incubation (37 °C, 200 rpm, 4 hr). The bacterial cell pellet was resuspended in 20 mM Tris buffer (pH 8) with 1 cOmplete protease inhibitor tablet (Roche). Following freeze-thawing at -20 °C, the cells were lysed by sonication. The soluble fraction of the lysate was separated from the cell debris by centrifugation (20,000 rpm, 20 min, 4 °C) and boiled (95 °C, 10 min) to precipitate impurities (αS remains soluble). Precipitated proteins were discarded after centrifugation (18,500 xg, 20 min, 4 °C), and ammonium sulfate was added to the supernatent create a 30% solution which was gently agitated (RT, 1 hr) to precipitate the protein (including αS). The precipitated protein was collected by centrifugation (18,500 xg, 20 min, 4 °C) and resuspended with gentle agitation in 20 mM Tris buffer (pH 8, 4 °C). The protein was purified by anionic exchange chromatography on an ÄKTA pure purification system (GE Healthcare) with a 5 mL HiTrap Q HP (GE Healthcare) pre-packed column. Fractions containing the purified protein were combined and further purified by size exclusion chromatography (SEC) using a HiLoad 16/60 Superdex 75 pg (GE Healthcare) pre-packed column and buffer exchanged into the experimental buffer (20 mM sodium phosphate buffer (pH 6.5)). αS eluted between 54 - 64 mL. The protein was aliquotted and flash frozen in liquid nitrogen, and stored at -80 °C until required.

Concentration of purified αS was determined by UV (280 nm) using a 2 mm quartz cuvette and an excitation coefficient (ε) of 4836 M^-1^cm^-1^. Purity of the eluted protein was confirmed by SDS-PAGE, and the correct product was confirmed via mass spectroscopy using an Agilent QTOF (ESI-QTOF) mass spectrometer. Circular dichroism (CD) spectral scan was used to confirm that the monomeric stock solutions of αS were random coil.

## **Preparation of lipid (DMPS) vesicles**

Suspension of 1,2-dimyristoyl-sn-glycero-3-phospho-L-serine (sodium salt) (DMPS) in 20 mM sodium phosphate buffer (pH 6.5, 2 mM) was incubated on a Thermomixer compact (Eppendorf) shaker (45 °C, 1400 rpm, 3 hr). The solution frozen and thawed five times using dry ice (15 minutes) and a Thermomixer compact (Eppendorf) shaker at 45 °C (0 rpm, 5 minutes). Lipid vesicles of the desired size were formed via sonication (Soniprep 150 plus sonicator, amplitude 10, 5 x 30 s with 30 s rest between rounds). Lipid concentration used is the monomer equivalent concentration. Their size distribution was measured by DLS, using a Zetasizer Nano ZSP (Malvern Instruments), to ensure a final consistent size of between 30 and 40 nm was obtained (Meade, RM., Watt, K.J.C., Williams R.J., and Mason, J.M. The Library Derived 4554W Peptide Inhibits Primary Nucleation of α-Synuclein J. Mol. Biol., 2021, 433, 167323). SUVs were chosen over LUVs since αS preferentially binds to SUVs over LUVs due to the high curvature and increased phospholipid packing defects. SUVs are~ 40 nm diameter; comparable in size to vesicles at the synaptic terminal of dopaminergic neurons. See Middleton, E. R.; Rhoades, E. Effects of Curvature and Composition on α-Synuclein Binding to Lipid Vesicles. Biophys. J. 2010, 99, 2279–2288.

## **Measurement of aggregation kinetics using lipid vesicles**

Solutions containing monomeric αS (100 µM), peptide (100 μM), DMPS vesicles (200 µM), ThT (50 µM) and sodium azide (0.01%) in 20 mM sodium phosphate buffer (pH 6.5) were prepared in a half-area 96-well nonbinding plate (Corning 3881), sealed with aluminium Thermowell sealing tape (Corning 6570), and incubated in a CLARIOstar plate reader (BMG Labtech) for up to 100 hours at 30 °C under quiescent conditions. Under these conditions, primary nucleation has been shown to be the predominant mechanism of aggregation (Meade, RM., Watt, K.J.C., Williams R.J., and Mason, J.M. The Library Derived 4554W Peptide Inhibits Primary Nucleation of α-Synuclein J. Mol. Biol., 2021, 433, 167323). Samples had a volume of 100 µL. Readings were taken at 1200 s intervals; λex = 440-10 nm, and λem = 480-10 nm, gain = 800, focal height = 4.9 mm. Each experiment was carried out in triplicate, error bars represent standard error.

## **Measurement of aggregation kinetics using shaking**

Solutions containing monomeric αS (300 µM), labelled-peptides (300 μM) and ThT (150 µM) in 20 mM sodium phosphate buffer (pH 6.5) were prepared in a half-area 96-well nonbinding plate (Corning 3881), sealed with aluminium Thermowell sealing tape (Corning 6570), and incubated in a CLARIOstar plate reader (BMG Labtech) for up to 100 hours at 37 °C with continuous shaking at 700 rpm (double orbital). Samples had a volume of 100 µL. Readings were taken at 360 s intervals; λex = 440-10 nm, and λem = 480-10 nm, gain = 800, focal height = 4.9 mm. Each experiment was carried out in triplicate, error bars represent standard error.

## **Aged-αS peptide spectra assay**

Aliquots of αS (500 μM, 20 mM sodium phosphate buffer, pH 6.5) were aged by incubating under aggregation conditions (700 rpm, 37 °C, Thermomixer compact (Eppendorf)) for up to 24 hr. To the aged αS (50 μM), labelled-peptide (25 μM) was added in a half-area 96-well nonbinding plate (Corning 3881), sealed with aluminium Thermowell sealing tape (Corning 6570), and the resulting fluorescence spectra was recorded (CLARIOstar plate reader, λ_ex_ 350 nm, λ_em_ 380 - 600 nm, gain 1800, focal height 4.2 mm, 37 °C). For continuous spectra readings, spectra were recorded every hour. Data was analysed using the Clariostar Data Processor python script.^292^

## **Fluorescence polarization**

Solutions containing αS (200 μM) and labelled-peptide (10 μM) in 20 mM sodium phosphate buffer, pH 6.5 were prepared in half-area 96-well non-binding plate (Corning 3881), sealed with clear sealing film, and incubated in a CLARIOstar plate reader (BMG Labtech) for 100 hr at 37 °C with agitation (700 rpm, double orbital). Samples had a volume of 100 μL and a final concentration 1% (v/v) DMSO. Readings were taken at 1 hr intervals, λex 360/20 nm and λex 530/40 nm filters, gains were: A=1599 and B=1623, and focal height of 3.8 mm. Each experiment was carried out in triplicate, the results plotted as the average change in milli-polarization (∆mP), using the reading from 1 hr as the baseline.

Fluorescence polarization (P) is calculated using the following equation:

P = (F|| - F⊥)/(F|| + F⊥)

Where F|| is the intensity of the parallel fluorescence emission and F⊥ is the intensity of the perpendicular fluorescence emission.

## **Continuous growth ThT experiment**

Solutions containing αS (200 μM) and ThT (100 μM) in 20 mM sodium phosphate buffer (pH 6.5) were prepared in a half-area 96-well nonbinding plate (Corning 3881), sealed with clear sealing film, and incubated in a CLARIOstar plate reader (BMG Labtech) for 100 hr at 37 °C with agitation (700 rpm, double orbital). Samples had a volume of 100 μL and a final concentration of 1% (v/v) DMSO. Readings were taken at 1 hr intervals, λex 450 nm, λex 482 nm, gain 900, and focal height 3.8 mm. Each experiment was carried out in triplicate, the results are plotted as the average change in fluorescence intensity (∆FI), using the reading from 1 hour as the baseline.

**Quantification of αS**

Following the end point of the ThT assays, the distribution of oligomer formation and modulation of the peptides was assessed using PICUP and SDS-PAGE. Shown in Figure S2, it is clear that all of the labelled-peptides significantly reduce oligomer formation compared to the unlabelled peptide (A10; 4554W). This is in line with the ThT results.

### **PICUP cross-linking SDS-PAGE**

Photo-induced cross-linking of unmodified proteins (PICUP) reactions were modified from a previously published protocol.^[37](https://www.sciencedirect.com/science/article/pii/S002228362100560X#b0185)^ Briefly, 20 μL of the end point (20 h) of the lipid-induced primary nucleation assay reaction mixture (100 μM αS, 50 μM ThT, 100 μM DMPS, 0–1000 μM peptide in 20 mM sodium phosphate buffer pH 6.5) was placed in a 1.5 mL Eppendorf tube. 2 μL of 1 mM solution of tris(2,2′bipyridyl)dichloro-ruthenium(II) hexahydrate (Ru(bpy)) in 20 mM sodium phosphate buffer pH 6.5, and 2 μL of 20 mM ammonium persulphate (APS) in 20 mM sodium phosphate buffer pH 6.5 were added to all samples simultaneously (by pulsing with a desktop centrifuge). The samples were then irradiated with ambient light for 10 s, and the reaction quenched with 10 μL RunBlue LDS Sample Buffer 4X concentrate (Expedeon). The samples were then heated to 95 °C for 5 min and fractionated by SDS-Page using a 12% Tricine RunBlue SDS Gel (Expedeon), and RunBlue run Buffer (Expedeon). The protein bands were visualised using Instant Blue (Expedeon) Coomassie stain. To analyse the gel band intensities the computer program ImageJ was used (<http://imagej.nih.gov/ij>). The image was first converted to grey scale and the raw integrated density (RID) of each band was measured with ImageJ. The data was then processed in excel to give a relative percentage of the band intensities.


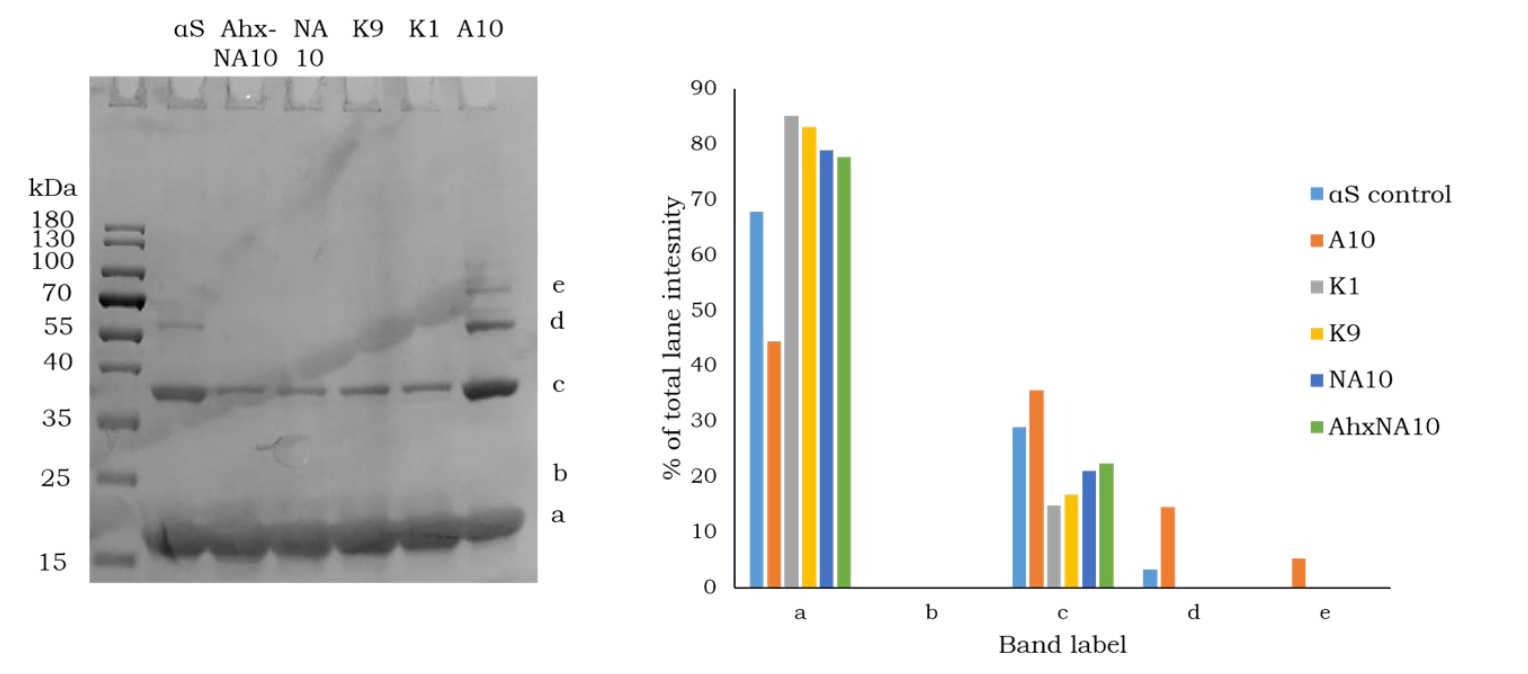


**Figure S2:** PICUP of peptides. Left) SDS-PAGE after PICUP has been preformed of the ThT end
point samples. Right) Analysis of the PICUP SDS-PAGE gel using ImageJ to analyse the relative ratio of the intensity of each band for each sample.
